# Supplementary material for: A qualitative study of stressors faced by older stroke patients in a convalescent rehabilitation hospital
Source: PLoS One. 2024 Aug 26;19(8):e0309457. doi: 10.1371/journal.pone.0309457 (PMC11346951; doi:10.1371/journal.pone.0309457)
Supplement: Attachment — Submitted filename: Response_to_Reviewers.docx [file pone.0309457.s002.docx]

**Response to reviewers**

**Reviewer #1**

***I found that this manuscript needs major revisions for publication in PLOSONE. This manuscript should follow the Consolidated Criteria for Reporting Qualitative Research (COREQ): a 32-item checklist for interviews and focus groups.***

**Response**

We thank the reviewer for highlighting this issue. We have made revisions to the manuscript following the Consolidated Criteria for Reporting Qualitative Research (COREQ) guidelines. We request the reviewer to check the revised manuscript.

**Materials and methods section**

**Line 71-72 in Preparation for the study**

The first author (hereafter, “the author”) is a M.S. student in comprehensive human sciences and male occupational therapist with six years of clinical experience in recovery rehabilitation.

**Line 87-95 in Interview procedure**

Three interviews were conducted between June and November, 2022. The interviewer asked questions according to an interview guide. Semi-structured in-person interviews were conducted in a private room in the hospital that the author is affiliated with, involving the patients and interviewer only. The first interview was conducted at the time of hospital admission, and subsequent interviews were conducted several times, with a gap of approximately one month. The interviews were recorded with the participants’ consent using the voice recorder function of an iPad and transcribed afterwards. The interview transcripts were not returned to participants for comments or correction. The interviewer recited the patients’ statements to them and made efforts to confirm the content of the statements to ensure data accuracy.

**Line 114–116 in Data analysis**

The data analysis and confirmation process were conducted by the author and three other authors who were not involved in the interview process.

**Reviewer #2**

***I want to congratulate the authors with this interesting study. However, the are several concerns that need be addressed before publication.*** ***Abstract: The authors state: This study yields valuable insights that contribute to a deeper 33 comprehension of the specific stressors experienced by older patients with stroke during the recovery process. It is however unclear what these insights are. It would be valuable to give some insights in the results of this study so clinicians can decide if this study is useful for them***

***Introduction line 40: please elaborate on the mental health problems.***

***Introduction line 41: Which adverse effects do you mean? It is important to be a bit more specific.***

***Line 58: please state the aim of the study more clearly***

***Page 5 line 61: start with describing the design of the study.***

***Page 5 line 67: please provide a reference for SCAT***

***Page 6 line 92: I think a strength of this study is the methodological approach (use of SCAT). It might be helpful to also state this in the abstract. The use of this approach might justify the low sample size.***

***Results: The results section is very short. The authors should provide more demographic information about the participants and also report quotes from the participants.***

***Discussion: The results of this study are based on only 4 participants. Therefore, more information should be presented in the results section. These might also lead to a more profound discussion section. Also, the strengths and limitations should be described more thoroughly. Why did the authors chose this design and analysis? What are the consequences of the limited sample size etc.***

**Response**

Thank you for the numerous suggestions. We realized that there were several points that needed clarification, including the purpose of the study, the reasons for adopting the analytical method, and the study limitations. Based on your feedback, we have made the following amendments to the manuscript.

**Abstract**

**Line 26–35**

The interviews were analyzed using the Steps for Coding and Theorization method for qualitative data analysis. The results of the qualitative analysis demonstrated that patients experienced specific stressors, such as, difficulty in movement of the paralyzed hand, fear of stroke recurrence, and dietary problems. Some stressors were manageable through healthcare professionals’ active and sensitive communication strategies. These stressors were derived from the theoretical framework of “stressors related to hospitalization” and “stressors related to the illness.” Additional stressors emerged from the interaction between these two types within the theoretical framework. The results of this study contribute to a deeper understanding of the specific stressors experienced by older stroke patients during the recovery process.

**Introduction section**

**Line 38–43**

Stress is a nonspecific response of the body to external stimuli [1]. Stress varies as the stressors faced by individuals differ depending on their age, sex, and social role [2]. Stressors include physical, biological, chemical, psychological, and social factors. The accumulation of these stressors causes stress, which, if not adequately addressed, can lead to physical or mental health problems, such as cardiovascular disease and depression, respectively [3]. To prevent these adverse effects, it is imperative to identify and address the stressors. To prevent these stress-related diseases, it is imperative to identify and address the stressors.

**Line 59–61**

The purpose of this study is to provide a deeper understanding of the specific stressors experienced by older stroke patients in convalescent rehabilitation wards during their hospital stay.

**Materials and methods section**

**Line 64–69**

We conducted a qualitative study and interviewed each participant separately. The interview transcripts were analyzed according to the “Steps for Coding and Theorization” method (SCAT), a sequential and thematic qualitative data analysis technique [10-12].

This study was conducted in accordance with the Consolidated Criteria for Reporting Qualitative Research (COREQ), a checklist designed to improve the transparency and reliability of qualitative research [13] (S1 Table).

**Line 72-75 in Preparation for the study**

Before this study was conducted, the author reviewed the literature on SCAT, conducted an analysis, and attended a workshop for SCAT developers to deepen his understanding of the analysis methods to ensure the accuracy of the analysis [10-12].

**Line 103–106 in Data analysis**

We predicted that the outcome of the interviews would be strongly influenced by the participants’ individual characteristics. Therefore, to obtain objective results, we used the SCAT technique that specializes in coding and theorization and can be applied to a small amount of data. The SCAT method consists of the following steps [10-12]:

**Results section**

**Line 132–133 in Storyline and theoretical descriptions**

In the sections below, the storylines and theoretical descriptions as well as quotes from each participant, are described.

**Line 144–146 in Case 1: Mr. A, facing an inconvenient situation**

*“Not being able to do things freely is the biggest stressor. All in all, there's nothing better than that. I can't eat what I like, or drink a lot. Even if I have a computer, I can't use my right hand. I can't even do my own hobbies. And, it is still significant whether or not you have a wife nearby.”*

**Line 154–156 in Case 1: Mr. A, facing an inconvenient situation**

*“The most important thing is the food. Anyway, there's nothing to do, so at least a meal, you'd think, wouldn't you? The food is different from when we're at home. It doesn't help that I can't walk. And, I think it's a bit hard not to have hobbies.”*

**Line 163-166 in Case 1: Mr. A, facing an inconvenient situation**

*“Whatever I do, my hands don't work. For example, when you brush your teeth. It's the same when you go to the toilet and wipe your bottom. I can't use my right hand. Also, I like books and I want to read, but I can't turn the pages. And, unlike in the past, I eat rice and side dishes every day. My eating habits have changed drastically.”*

**Line 175-178 in Case 2: Ms. B, facing stressors caused by communal living**

*“I sometimes have trouble sleeping well at night because of noises or brightness. Everyone is trying to go to the toilet before rehabilitation, so the timing is… And with male nurses, there was a bit of resistance to using the toilet. After all, in shared living arrangements, everyone has a different rhythm of life.”*

**Line 188-192 in Case 2: Ms. B, facing stressors caused by communal living**

*“Like last time, in shared living arrangements, everyone has a different rhythm of life, but it can't be helped. Sleep, you know, because some people go to the toilet at night or early in the morning, so it's quite noisy and you can't sleep well. And the nurse puts the patient next to me to sleep, and there are all sorts of noises when she does that. We all have the same desire to go to the toilet before rehabilitation, so we don't make it in time. Toilets are a perpetual problem.”*

**Line 201-203 in Case 2: Ms. B, facing stressors caused by communal living**

*“Like how to communicate with people in the room. Like sleeping. Because of the lights and noise when my roommate goes to the toilet at night. Roommates have different living patterns. In rehabilitation, though, there were some questionable things like time changes.”*

**Line 212-214 in Ms. C, facing an excrement problem and anxiety about stroke recurrence**

*“I don't like the situation of one toilet for four people. I and others are suffering. I thought it was hard.* *I didn't know that you have to press the nurse call. Then I wished they had told me from the beginning.* *That was a bit of a shock.”*

**Line 221-223 in Ms. C, facing an excrement problem and anxiety about stroke recurrence**

*“I feel bad because I have to go to the toilet in the middle of the night. But I try to be patient.* *If it was during the day, I would ask the nurse to help me, but at night I would still feel sorry.* *It's painful. You have to be experienced to understand.”*

**Line 231-233 in Ms. C, facing an excrement problem and anxiety about stroke recurrence**

*“I have a little bit of anxiety about my future and my life. Because I've got comfortable here. And I don't know what I would do if I fell ill again.* *No one is going to talk to me about it. I'm a bit worried about that. That's what I'm most worried about.”*

**Line 240-241 in Case 4: Mr. D, facing a meal problem**

*“I've had a leak before the nurse came. She can't come right away, she's too busy.* *It's gone now.”*

**Line 247-250 in Case 4: Mr. D, facing a meal problem**

*“The rice and side dishes are cold. So I feel sorry to leave it.* *I can eat it beautifully when it's warm. But when it's cold, I just can't.* *After the meal, I can't wash my hands because some people wash their hands in their rooms first.* *When I want to go into the toilet, there are people ahead of me. It can't be helped.”*

**Line 256-257 in Case 4: Mr. D, facing a meal problem**

*“Side dish is cold. Wish it was room temperature.* *I eat my meals late, so I'm the last one to go back to my room.* *So, I can't wash my hands first.”*

**Discussion section**

**Line 339–351**

Limitations

In conclusion, we clarified the stressors faced by older stroke patients in convalescent for rehabilitation. However, this study has some limitations. First, the study was severely limited by the small number of stroke patients, which prevents us from drawing some important conclusions. The SCAT method can be used to analyze data from a small number of people because it provides a theoretical description from the participants’ storylines; however, the number of participants in this study was not sufficient to generalize the findings. Second, this study did not fully consider the participants’ individual characteristics, such as personality and background, nor did it analyze the patients in terms of their pathology and sequelae. Therefore, the results obtained should be interpreted carefully, as individual bias was not sufficiently eliminated. In future, it is necessary to select other participants and data analysis methods that consider participants’ individual characteristics and the aftereffects of stroke and recruit more participants to elucidate the stressors faced by older stroke patients in convalescent rehabilitation.

**Conclusion section**

**Line 358–362**

To the best of our knowledge, thus far, no reports have identified the specific stressors faced by older stroke patients. Therefore, this study provides valuable information from a first-hand perspective that will lead to a deeper understanding of the specific stressors experienced by older stroke patients during recovery.

**References**

1. Selye H. Forty years of stress research: principal remaining problems and misconceptions. Can Med Assoc J. 1976;115: 53-56.

3. Schneiderman N, Ironson G, Siegel SD. Stress and health: psychological, behavioral, and biological determinants. Annu Rev Clin Psychol. 2005;1: 607-628. doi: 10.1146/annurev.clinpsy.1.102803.144141

10. Otani, T. (2008) SCAT a Qualitative Data Analysis Method by Four-Step Coding: Easy Startable and Small Scale Data-Applicable Process of Theorization (in Japanese with English Abstract). Educational Sciences, 54, 27-44.

11. Otani T. Qualitative research series SCAT: Steps for Coding and Theorization— an explicit procedure for qualitative data analysis that is easy to undertake and applicable to small data Journal of Japan Society of Kansei Engineering. 2011;10: 155-160.

12. Otani T. Paradigm and design of qualitative study: from research methodology to SCAT. Nagoya: The University of Nagoya Press; 2019. (in Japanese)

**Supporting information section**

**Line 458-460**

Supporting information

S1 Table. Consolidated criteria for reporting qualitative studies (COREQ): a 32-item checklist.

**S1 Table. Consolidated criteria for reporting qualitative studies (COREQ): a 32-item checklist**

Developed from:

Tong A, Sainsbury P, Craig J. Consolidated criteria for reporting qualitative research (COREQ): a 32-item checklist for interviews and focus groups. International Journal for Quality in Health Care. 2007. Volume 19, Number 6: pp. 349 – 357.

| **No. Item** | **Guide questions/description** | **Reported on Page #** |
| --- | --- | --- |
| **Domain 1: Research team and reflexivity** | | |
| *Personal Characteristics* | | |
| 1. Interviewer/facilitator | Which author/s conducted the interview or focus group? | Page 5 |
| 2. Credentials | What were the researcher’s credentials? *E.g. PhD, MD* | Page 5 |
| 3. Occupation | What was their occupation at the time of the study? | Page 5 |
| 4. Gender | Was the researcher male or female? | Page 5 |
| 5. Experience and training | What experience or training did the researcher have? | Page 5 |
| *Relationship with participants* | | |
| 6. Relationship established | Was a relationship established prior to study commencement? | Page 6 |
| 7. Participant knowledge of the interviewer | What did the participants know about the researcher? *e.g. personal goals, reasons for doing the research* | Page 6 |
| 8. Interviewer characteristics | What characteristics were reported about the interviewer/facilitator? *e.g. Bias, assumptions, reasons and interests in the research topic* | Page 5 |
| **Domain 2: study design** | | |
| *Theoretical framework* | | |
| 9. Methodological orientation and Theory | What methodological orientation was stated to underpin the study? *e.g. grounded theory, discourse analysis, ethnography, phenomenology, content analysis* | Pages 6-7 |
| *Participant selection* | | |
| 10. Sampling | How were participants selected? *e.g. purposive, convenience, consecutive, snowball* | Page 5 |
| 11. Method of approach | How were participants approached? *e.g. face-to-face, telephone, mail, email* | Pages 5-6 |
| 12. Sample size | How many participants were in the study? | Page 8 |
| 13. Non-participation | How many people refused to participate or dropped out? Reasons? | Page 8 |
| *Setting* | | |
| 14. Setting of data collection | Where was the data collected? *e.g. home, clinic, workplace* | Page 6 |
| 15. Presence of non-participants | Was anyone else present besides the participants and researchers? | Page 6 |
| 16. Description of sample | What are the important characteristics of the sample? *e.g. demographic data, date* | Pages 5-7 |
| *Data collection* | | |
| 17. Interview guide | Were questions, prompts, guides provided by the authors? Was it pilot tested? | Page 6 |
| 18. Repeat interviews | Were repeat interviews carried out? If yes, how many? | Pages 6-7 |
| 19. Audio/visual recording | Did the research use audio or visual recording to collect the data? | Page 6 |
| 20. Field notes | Were field notes made during and/or after the interview or focus group? | Page 6 |
| 21. Duration | What was the duration of the interviews or focus group? | Page 6 |
| 22. Data saturation | Was data saturation discussed? | Pages 20-21 |
| 23. Transcripts returned | Were transcripts returned to participants for comment and/or correction? | Page 6 |
| **Domain 3: analysis and findings** | | |
| *Data analysis* | | |
| 24. Number of data coders | How many data coders coded the data? | Page 7 |
| 25. Description of the coding tree | Did authors provide a description of the coding tree? | NA |
| 26. Derivation of themes | Were themes identified in advance or derived from the data? | Pages 8-15 |
| 27. Software | What software, if applicable, was used to manage the data? | NA |
| 28. Participant checking | Did participants provide feedback on the findings? | No, not specifically. |
| *Reporting* | | |
| 29. Quotations presented | Were participant quotations presented to illustrate the themes / findings? Was each quotation identified? *e.g. participant number* | Pages 9-15 |
| 30. Data and findings consistent | Was there consistency between the data presented and the findings? | Pages 9-15 |
| 31. Clarity of major themes | Were major themes clearly presented in the findings? | Pages 15-19 |
| 32. Clarity of minor themes | Is there a description of diverse cases or discussion of minor themes? | Pages 9-15 |
